# Functional near‐infrared spectroscopy approach to the emotional regulation effect of drawing: Venting versus distraction

**DOI:** 10.1002/brb3.3248

**Published:** 2023-09-12

**Authors:** Xinlei Zhang, Wenhua Yan, Cheng Xu, Aiping Yang, Zihan Shen, Xinwei Guo

**Affiliations:** ^1^ School of Psychology and Cognitive Science East China Normal University Shanghai China; ^2^ Shanghai Key Laboratory of Mental Health and Psychological Crisis Intervention, School of Psychology and Cognitive Science East China Normal University Shanghai China

**Keywords:** distraction, drawing, emotion regulation, fNIRS, venting

## Abstract

**Background:**

Drawing can regulate emotions through venting or distraction. Distraction is more helpful for short‐term emotion recovery; however, the sustainability of this difference is yet to be clarified. This study used functional near‐infrared spectroscopy (fNIRS) to explore potential differences between venting and distraction.

**Methods:**

A total of 44 college students participated in the experiment. After inducing fear by video, they were divided into two groups: The venting group drew their emotional experience, and the distraction group drew a house. Subsequently, the participants were instructed to relax by a brief video.

**Results:**

Although the distraction group had a higher valence than the venting group at the end of the drawing activity, there was no difference between the two groups after a relaxation period. Additionally, the activation pattern of the prefrontal cortex differed between the two groups. Compared to the distraction group, the venting group had fewer channels with elevated prefrontal activity during drawing, suggesting less cognitive control, and had more channels with reduced prefrontal activity during relaxation, suggesting a higher level of relaxation. Drawing coding and fNIRS data were both associated with variations in valence.

**Conclusion:**

The less the cognitive control over emotion and the more free the expression of emotion during drawing, the higher the increase in valence; inversely, the more the cognitive control over emotion and the less free the expression of emotion, the lower the increase in valence.

## Graphical abstract

Compared to drawing to distract fear, drawing to express fear had a smaller region in the prefrontal cortex with elevated activity, suggesting less cognitive control, and had a larger region in the prefrontal cortex with reduced activity during relaxation after drawing, suggesting a higher level of relaxation.

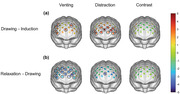

As an art‐making activity, drawing can improve mood by sublimating and releasing negative emotions. Therefore, it has been widely used in art therapy. Outside of art therapy, laboratory studies have also found that art‐making can be used as a form of emotion repair for nonclinical participants. Art‐making involves a variety of emotion regulation strategies, but two are most closely associated with art‐making: venting (catharsis or expression) and distraction (redirection) (Drake & Winner, 2012; Gruber & Oepen, 2018). Venting is the expression of strong emotions through drawing to release confined emotions. This strategy can be traced back to Freud's theories (Vives, 2011; Freud & Breuer, 1895/1955). Distraction involves shifting attention from negative thoughts and feelings by focusing on something else. Creating something positive or neutral would lead people away from the rumination of negative feelings to a more positive emotional state (De Petrillo & Winner, 2005).

Placing these two types of emotion regulation strategies into the larger framework of emotion regulation can help us understand their respective roles. Gross (2002) proposed a process model of emotion regulation based on the location of various emotion regulation strategies in the emotional process. It categorizes emotion regulation strategies into situation‐, cognition‐, and response‐focused strategies. Situation‐focused strategies are at the first stage and refer to seeking, avoiding, or changing emotion‐inducing situations (Gross, 2001). However, when people are unable to control the situation themselves, or when an unpleasant situation has already occurred, cognition‐focused strategies allow people to focus on one aspect of the situation or ignore others or to interpret the meaning of the situation in a less distressing way. The final stage is the response‐focused strategy, which does not change the situation or the appraisal of the situation but rather changes the feeling or expression of the emotion. Apparently, distraction belongs to the cognition‐focused strategy, whereas venting belongs to the response‐focused strategy.

Several studies have compared the effectiveness of different emotion regulation strategies. In general, situation‐focused strategies have been found to be more effective (Ong et al., 2005): If people can avoid or minimize the problem, no unpleasant situation arises in the first place. However, situation‐focused strategies are not suitable for all situations; cognition‐ and response‐focused strategies each have their own adaptive situations. In studies of emotion regulation through art‐making, the comparison between venting and distraction strategies has been the focus of attention.

## 1 EMOTION REGULATION EFFECTS OF VENTING AND DISTRACTION IN ART‐MAKING

De Petrillo and Winner (2005) presented participants with tragic photos with themes related to illness, death, and poverty and asked the experimental group to draw based on their feelings, whereas the control group performed non‐drawing activities (copying shapes or completing word puzzles). It was found that there was a greater increase in valence (as measured by the Affect Grid) after drawing in the experimental group than in the control group. Moreover, categorizing the content of the drawings revealed that participants who created negative drawings (i.e., using the venting strategy) had the same degree of emotional improvement as those who created nonnegative drawings (i.e., using the distraction strategy), suggesting that both venting and distraction are effective emotion regulation mechanisms in art‐making.

Subsequent research has found that when drawing is used as a distraction, it appears to be more effective than venting in improving negative emotions. In a study conducted by Dalebroux et al. (2008), participants were divided into three groups after inducing negative emotions. Those in the venting group created a drawing expressing the current emotion. Those in the positive emotion group created a drawing depicting something happy. Those in the control group circled specific symbols on paper. The results revealed that the positive emotion group showed the greatest improvement in mood valence (as measured by the Affect Grid), whereas the venting and control groups showed no significant difference before and after the task.

Further research has found that the content of distraction drawings does not need to be positive. Creating neutral drawings can also improve emotion. Drake and Winner (2012) replaced the positive emotion group with a distraction group and asked participants to create emotionally neutral objects (houses). They found that the distraction group still had greater improvement in both positive and negative affect (as measured by the Positive and Negative Affect Schedule) than the venting and control groups. Diliberto‐Macaluso and Stubblefield (2015) divided participants into four groups after inducing angry emotions: a venting group (drawing what they are feeling at the moment), positive distraction group (drawing something that makes them feel happy), neutral distraction group (drawing a still life), and control group (completing a word search puzzle). The results revealed that the positive and neutral distraction groups showed higher improvement in valence (as measured by the Affect Grid) than the other two groups.

Although literature in this field is scarce, some research trends remain visible. The first is to expand the types of induced emotions. Early research focused on inducing sadness; however, there may be differences between emotions, and it is necessary to examine whether existing findings can be generalized to other emotions. For example, Genuth and Drake (2021) compared the effects of using drawing to regulate sadness and anger and found that regardless of the emotion induced (sadness or anger), participants in the distraction group experienced greater mood improvement (as measured by the Positive and Negative Affect Schedule) than those in the venting group. These results suggest that the findings on sadness can be generalized to anger.

The second is to expand the forms of art‐making. Smolarski et al. (2015) divided participants into a positive expression group (expressing happiness through drawing), venting group (expressing current stress through drawing), and distraction control group (tracing and coloring a line drawing of a sailboat) after inducing stress. The results showed that negative mood (as measured by the Profile of Mood States) improved significantly in all three conditions, but the positive expression group showed greater improvement than the other two groups. Forkosh and Drake (2017) compared the effects of coloring and drawing on the regulation of sadness. After inducing sadness, they randomly assigned participants to the coloring group (coloring a mandala design), drawing group (drawing a design), and drawing‐to‐express group. The results revealed that the positive affect (as measured by the Positive and Negative Affect Schedule) was significantly improved in both distraction conditions (the coloring and drawing groups) and was higher than in the drawing‐to‐express group. However, there was no difference in the change in negative affect among the three groups. Similarly, Turturro and Drake (2022) replaced sadness with anxiety and found that all three prior activities significantly decreased anxiety (as measured by the State‐Trait Anxiety Inventory), decreased heart rate, and increased respiratory sinus arrhythmia (RSA, which can be regarded as a physiological indicator of successful emotion regulation), with no between‐group differences.

The third is to compare art‐making with other activities. James et al. (2018) compared the differences in the emotion regulation effects of venting and distraction strategies in four activities: drawing, writing, talking, and thinking. The results showed that compared to venting, distraction increased the positive affect (as measured by the Positive and Negative Affect Schedule) in drawing and thinking activities. Meanwhile, distraction decreased the negative affect more than venting in all four activities. Overall, distraction is a more effective emotion regulation strategy than venting for improving emotions.

However, these findings may have limited insight into practice. Previous studies have examined only the immediate emotional state at the end of drawing activities, and it is easy to conclude that distraction is more effective than venting. This is consistent with the findings in the field of emotion regulation. For instance, people who are encouraged to cry while watching a sad movie feel sadder than those who restrain their emotions (Kraemer & Hastrup, 1988). Although those who restrain their emotions may not appear as sad as those who express them, according to Freud, these unexpressed emotions are repressed and not released (Freud & Breuer, 1895/1955). More importantly, in the practice of art therapy, expressing emotions enables the release of repressed energy and allows people to learn to face and process these experiences, prompting self‐awareness and changes (Coholic, 2011). Contrastingly, distraction implies avoidance. The long‐term use of distraction strategies can prevent people from taking steps to improve their situation (Wolgast & Lundh, 2017). Research has found a negative correlation between the habit of relying on distraction to regulate emotions and the level of mental health (Shiota, 2006).

## 2 INTRODUCTION OF FUNCTIONAL NEAR‐INFRARED SPECTROSCOPY IN ART THERAPY RESEARCH

Neuroimaging techniques to provide physiological indicators have gradually become an emerging trend in art therapy research. Although a large body of literature indicates the clinical value of art therapy, determining the mechanisms of the changes that occur during art therapy has been a challenge (King & Kaimal, 2019). Current neuroimaging techniques offer a viable way to measure brain activity in realistic settings. They can help elucidate how art‐making in the therapeutic relationship employs the brain and can provide scientific data for measuring the mechanisms of change and effects of clinical art therapy interventions (Kaimal, 2023; King & Kaimal, 2019; King et al., 2019).

Initial studies used electroencephalogram techniques (Belkofer et al., 2014; Belkofer & Konopka, 2008; King et al., 2017; Kruk et al., 2014). A common practice is to collect a segment of resting‐state signals before and after the experimental task to examine the effects of the task on brain waves. Recently, functional near‐infrared spectroscopy (fNIRS), an emerging brain imaging technique characterized by convenience, safety, and relatively low sensitivity to head movements (Zhu, 2020), has made it possible to record the entire drawing process.

Kaimal et al. (2017) contributed to the initial exploration of this field. They found that during coloring, doodling, and free drawing, the activity of the left medial prefrontal cortex (mPFC) was higher than that in the resting state. Because the mPFC is associated with rewards, they hypothesized that all three forms of drawing would enable participants to perceive rewards. However, the activity of the mPFC is influenced by many factors and does not correspond only to the level of reward, thus reducing the persuasiveness of this finding. Another study conducted by Kaimal et al. (2022) found that the copying task led to stronger prefrontal activity than the creative self‐expression task in virtual reality drawing, implying that the copying task requires focused attention. In contrast, creative self‐expression reduces prefrontal load and induces relaxation. Yan, Zhang et al. (2021) found that when participants drew after inducing sadness, the frontopolar and left dorsolateral prefrontal cortex (dlPFC) had lower activity than the baseline. Because the dlPFC is associated with the inhibition of negative emotions, it was hypothesized that participants reduced their attention to sadness during drawing.

There are also studies comparing differences in brain activity during drawing between patients with mental illnesses and healthy controls. Nakano et al. (2018) compared brain activity in patients with schizophrenia and healthy controls during a tree‐drawing test. They found that healthy controls had higher activation in the bilateral frontal pole and left inferior frontal region during free drawing than during copying. In contrast, patients with schizophrenia did not differ between the two tasks. Furthermore, the latter showed lower activity in several brain regions than healthy controls, suggesting that they have deficits in cognitive function. Yan, Ji et al. (2021) compared the difference in brain activity between patients with schizophrenia and healthy controls when expressing emotions through drawing. They found that people with schizophrenia had weaker brain activity than healthy controls do, and they concluded that patients with schizophrenia could not immerse themselves in emotion expression. However, because no control group was set, the findings could also be interpreted as follows: The low activity in patients with schizophrenia might not be due to the content of the drawing but rather to the drawing activity itself.

Studies using the fNIRS imaging technique also face certain problems. The first problem was the setup of the experimental conditions. In terms of form, art‐making includes coloring, copying, doodling, and drawing; in terms of content, there are drawings of specified themes as well as completely open‐ended (free) drawings. Individual studies only select certain types of tasks, and it is still inconclusive whether different forms and contents of art‐making trigger different intensities of brain activity. In addition, a variety of control conditions, such as calculating (Yan, Zhang et al., 2021), coin flipping, and pencil spinning (King et al., 2017), were set to contrast with the drawing. Nonetheless, they also introduced extraneous variables, such as task difficulty and body movement. A more critical challenge lies in the interpretation of the results. Most current brain imaging studies only subjectively interpret the results. However, without evidence to support a correlation between brain imaging indicators and other indicators (such as scale scores), it is unconvincing to conclude that brain imaging data reflect issues of interest, including attention, relaxation, or pleasure.

## 3 PROPOSAL OF THE PRESENT STUDY

This study aimed to use the fNIRS imaging approach to explore potential differences between the regulation effects of venting and distraction strategies in drawing the fear emotion, providing physiological evidence for the emotion regulation effect of art‐making. Previous experimental studies have demonstrated that distraction strategies are more helpful for short‐term emotion repair. However, they only measured emotional state immediately at the end of art‐making. The sustainability of this difference between venting and distraction is yet to be clarified. If we increase the delay time of the posttest, it is likely that the valence of the venting group would improve.

Following the procedure used in previous studies, the present study divided the participants into venting and distraction groups after emotion induction. It was hypothesized that the distraction group would have a higher valence than the venting group after drawing. In this study, a subsequent relaxation period was added. This was set not only for ethical considerations (to let participants relax after experiencing negative emotions) but also to examine whether the difference between the two groups would diminish or reverse after a short period.

Hypothesis 1 The valence of the distraction group is higher than that of the venting group at the end of drawing. However, at the end of the relaxation period, there is no difference in valence between the two groups.

Early studies mostly focused on sadness; therefore, it was necessary to examine how generalizable these findings are to other emotions. This study induced the emotion of fear. As fear is one of the most typical emotions (Fehr & Russell, 1984; Shaver et al., 1987), with clear feelings, specific assessments, unique facial and vocal expressions, strong physiological responses, and an obvious action tendency (freezing, flight, or fight when desperate) (Shiota & Kalat, 2018), the study of fear can help reveal the general rule of emotions. Regarding emotion induction methods, the present study used a clip from a frightening film. This is because the intensity of emotions induced by videos is relatively high and can be sustained for a certain period (Westermann et al., 1996).

We used fNIRS to collect brain activity data throughout the experimental procedure, providing relevant physiological indicators to self‐report scales and new evidence that the scales could not reveal. Based on previous literature, this study chose the prefrontal cortex as the brain region of interest. Most brain imaging studies on emotion regulation have focused on cognition‐focused strategies and have identified an essential role of the prefrontal cortex. Prefrontal activity is significantly elevated when participants are asked to suppress emotions or when cognitive reappraisal is performed (Beauregard et al., 2001; Goldin et al., 2008; Ochsner et al., 2002), and the prefrontal cortex is a brain region associated with cognitive control (Tisserand et al., 2004). A meta‐analysis conducted by Buhle et al. (2014) indicated that cognitive reappraisal activates domain‐general cognitive control areas, including the dorsal mPFC, dlPFC, ventral lateral prefrontal cortex (vlPFC), and posterior parietal lobe. Kohn et al. (2014) proposed a neural process model of emotion regulation, suggesting that the vlPFC is responsible for signaling the need for regulation, leading to the regulation of cognitive processes conducted by the dlPFC.

In conclusion, the degree of cognitive control reflected by the activation of the prefrontal cortex is closely related to emotion regulation. In the present experiment, if there were differences in participants’ self‐reported emotional states between the venting and distraction conditions, we expected that there would also be differences in the activation of brain regions related to emotion regulation. The distraction condition required participants to shift their attention from negative emotions, which required cognitive control by the prefrontal cortex, whereas the venting condition was relatively more straightforward.

Hypothesis 2 Prefrontal activity is higher in the distraction condition than in the venting condition.

In addition, a subjective report questionnaire on the content of the drawings was included in this study, which served as coding material alongside the drawings. The self‐report emotion scales, fNIRS data, and coding formed three sources of data for the same process, and we expected that they could produce a mutual corroboration relationship.

Hypothesis 3 The fNIRS data and coding of drawings are associated with self‐reported emotion states.

## 4 METHODS

### 4.1 Participants

A total of 44 college students were recruited as paid participants, including 13 males and 31 females, with an age range of 19–29 years (*M* = 22.39, *SD* = 2.74). All participants were right‐handed, in good health, and had no professional drawing training. The study was approved by the University Committee on Human Research Protection, and all participants signed informed consent forms.

### 4.2 Materials

#### 4.2.1 Drawing materials

**FIGURE 1:**
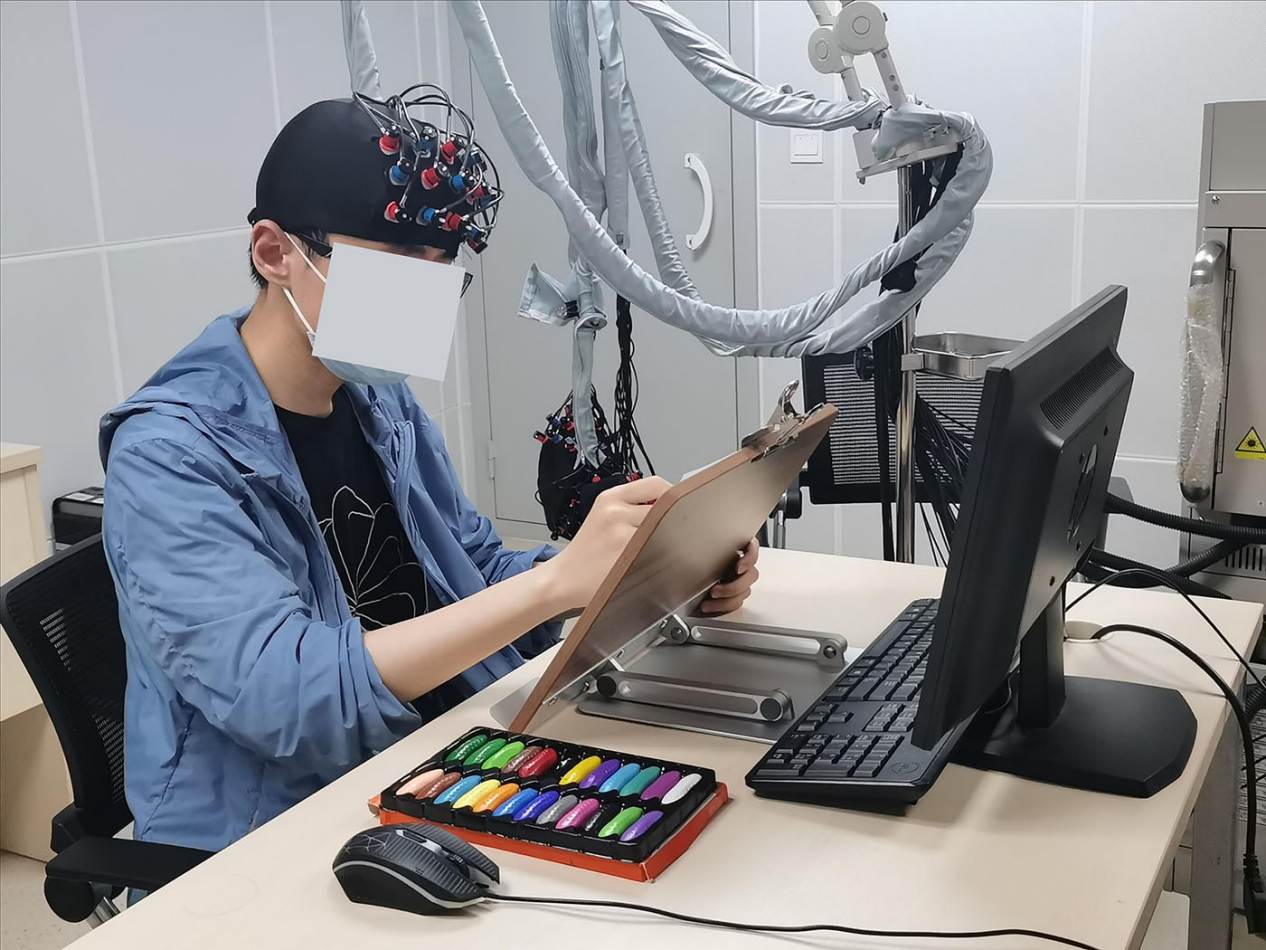
Laboratory settings.

The drawing materials included A4 white paper and 24 color oil painting sticks. These materials are common in art therapy, are easy to use and clean, and can promote expression (Belkofer et al., 2014). As brain activity data were also collected using fNIRS, A4 white paper was placed on a small holder at a certain angle to reduce the affect of head movement on the signals, as shown in Figure 1.

#### 4.2.2 Emotion induction video

A clip from the film, *Dead Silence*, was used to induce fear. Jin et al. (2009) found that this film clip could induce fear with high intensity and differentiation. The length of the original video was 2 min 17 s, but the preliminary experiment found that it induced an excessively high intensity of disgust. Therefore, the video was cut, and the length was reduced to 1 min 54 s to improve the differentiation degree of fear.

#### 4.2.3 Emotion relaxation video

An original emotion relaxation video was used to guide the participants to relax and calm their emotions, which lasted for 3 min 48 s. One researcher wrote and recorded instructions which followed the principles of relaxation training (Li, 2002), guiding participants into a relaxed state through deep breathing. The recording was equipped with a scenery clip and a piece of light background music, “Ocean (Pacific).”

#### 4.2.4 Scale and questionnaire

The Affect Grid developed by Russell et al. (1989) was used to measure the valence and arousal of emotions. The scale is a 9 × 9 two‐dimensional grid, with the horizontal axis from left to right representing unpleasant feelings—pleasant feelings, and the vertical axis from bottom to top representing sleepiness—high arousal. The participants were required to place one checkmark “×” somewhere in the grid according to their current emotional state. In the analysis, according to its coordinate position, the mark was converted into two indicators, valence and arousal, both of which were scored from 1 to 9. The higher the value, the higher the degree of valence or arousal.

An original subjective report questionnaire was used to collect descriptions of the drawing process and its content. The questionnaire consisted of six questions about the emotion while drawing, the content of the drawing, the association of the drawing, the relation between the drawing and real life, the relation between the drawing and the emotion while watching the video, and the naming of the drawing.

### 4.3 Procedure

**FIGURE 2:**
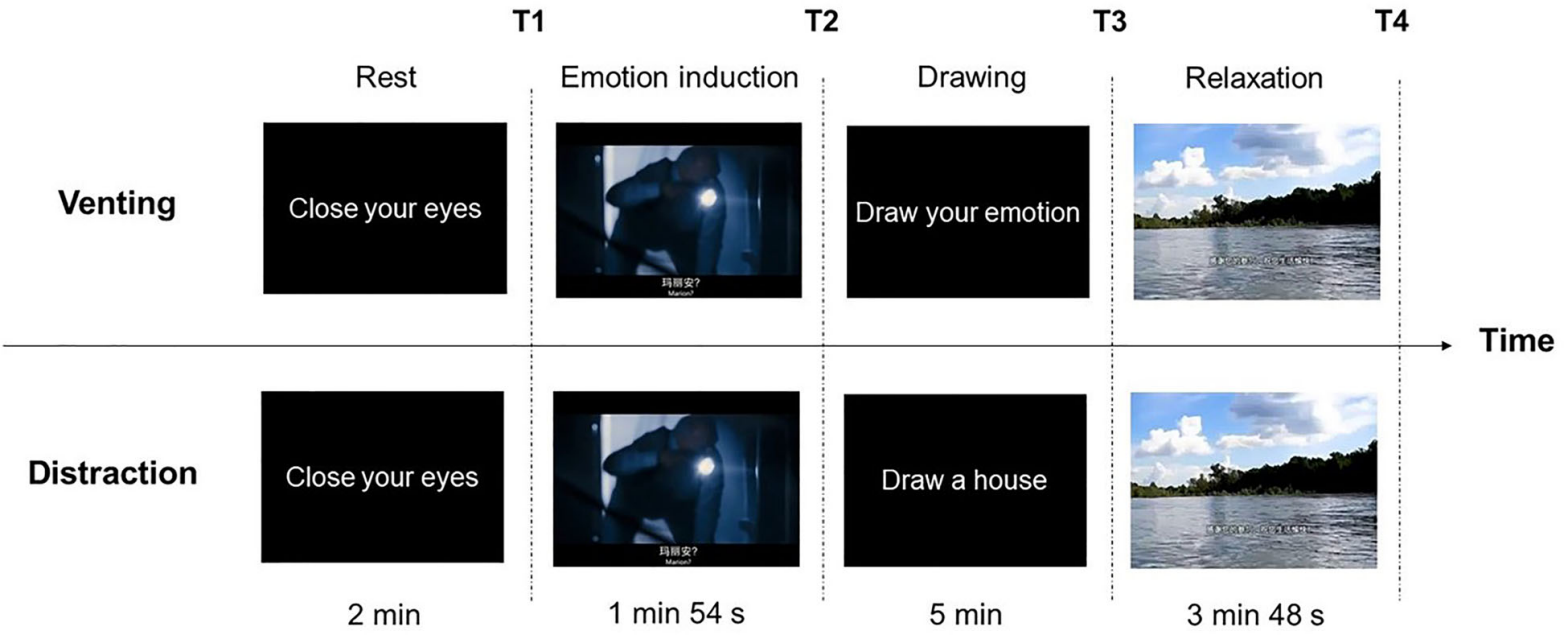
The experimental procedure. *Note*: The experimental procedure mainly included a 2‐min rest period with eyes closed, a 1 min 54 s fear induction period, a 5‐min drawing period, and a 3 min 48 s relaxation period. The difference between the two groups was in the drawing period, in which different instructions were presented on the screen. The venting condition required participants to express the emotions they had just experienced while watching the film, whereas the distraction condition required them to draw a house. The participants filled in the Affect Grid four times at *T*1–*T*4. At *T*3, in addition to the Affect Grid, the participants filled in the subjective report questionnaire.

The participants were divided into venting and distraction groups, with 22 participants in each group. There were no differences in sex across conditions, *χ* ^2^ = .109, *p* = .741. The fNIRS device, which monitored the activity of the prefrontal cortex, was worn at the beginning of the study and removed toward the end. The experimental procedure was presented by E‐Prime 2.0, which mainly included a 2 min resting period with eyes closed, a 1 min 54 s fear induction period, a 5 min drawing period, and a 3 min 48 s relaxation period. The experimental process is illustrated in Figure 2. For the venting group, the instruction presented in the drawing period was *Please draw on the white paper with the oil painting sticks in front of you, expressing the emotion you experienced when watching the film just now*. For the distraction group, the instruction was *Please draw a house on the white paper with the oil painting sticks in front of you*. The house is a neutral object that could distract participants from the negative emotion that the film induced, and “drawing a house” was adopted as the distraction task by previous studies (Drake et al., 2012). During the procedure, participants filled in the Affect Grid four times: before emotion induction (*T*1), after emotion induction (*T*2), after drawing (*T*3), and after relaxation (*T*4). At *T*3, in addition to the Affect Grid, the participants filled in the subjective report questionnaire. The statistical analysis software IBM SPSS Statistics 23 was used to analyze the experimental data.

### 4.4 fNIRS data collection and analysis

An fNIRS system (ETG‐7100, Hitachi Medical Corporation) was used to collect the fNIRS data. The device follows changes in hemoglobin concentration in the cerebral cortex by emitting and receiving near‐infrared light at two wavelengths, 695 and 830 nm, to infer neural activity in the brain.

**FIGURE 3:**
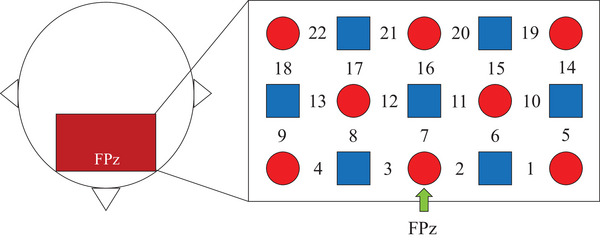
Probe patch placement. *Note*: The prefrontal cortex was covered with a probe patch with three rows and five columns, with the probe in the middle of the bottom row placed approximately at the FPz point. The red circles in the figure represent the emitter probes, and the blue squares represent the detector probes. Seven emitter and eight detector probes formed 22 recording channels (Channels 1–22).

The prefrontal cortex was covered with a probe patch with three rows and five columns, forming 22 recording channels (Channels 1–22), and the distance between the adjacent emitter probes and detector probes was 30 mm. The placement of the patch followed the international 10–20 reference system. Referring to previous studies (Yan, Zhang et al., 2021), the probe in the middle of the bottom row was placed approximately at the FPz point (see Figure 3).

The localization data were acquired using the VPen head 3D localization information visualization recording system, followed by probabilistic registration (Singh et al., 2005) to align the localization data to the standard space. The mean Montreal Neurological Institute (MNI) coordinate values for each channel are listed in Table 1.

**TABLE 1:** Mean Montreal Neurological Institute (MNI) coordinate values corresponding to each channel.

Channel	*X*	*Y*	*Z*
1	−36	63	2
2	−13	73	7
3	18	73	7
4	41	60	2
5	−46	51	10
6	−26	67	16
7	4	68	19
8	31	65	16
9	49	49	11
10	−35	56	23
11	−11	65	29
12	18	64	29
13	41	53	23
14	−44	40	32
15	−22	54	38
16	5	57	41
17	28	52	38
18	47	37	32
19	−34	39	43
20	−10	48	50
21	19	47	48
22	38	37	43

The NIRS‐KIT software package (Hou et al., 2021) based on MATLAB was used to analyze the fNIRS signal data. The main procedures were as follows: (1) data preprocessing, including first‐order detrending, head‐motion correction using the correlation‐based signal improvement method, and low‐pass filtering with an infinite impulse response filter at the upper limit of 0.08 Hz; (2) the general linear model was used to model the oxyhemoglobin data and calculate the hemodynamic response index of individuals at different periods; and (3) group statistical inference was performed using *α* = 5% as the significance threshold, and the false discovery rate criterion was used for correction.

### 4.5 Drawing coding methods

A coding approach was used to analyze the drawings and subjective reports of the participants (Yan, Ji et al., 2021). First, four researchers generated a coding manual after a thorough discussion based on the research purpose. The rationale behind the coding is to use both objective data (drawers' drawings) and subjective data (drawers' self‐expression), as well as researchers’ understanding of these data. The coding manual consisted of three sections: (1) color characteristics, (2) drawing content, and (3) literal description from the drawer, with 13 coding indicators, as shown in Table 2. As the participants in the distraction condition had already been assigned a theme, b2: “Distinctness of the theme” was a separate coding indicator for the venting condition. The coding of Part A was mainly performed using the color analysis tool *Image Color Summarizer*, and researchers only entered the number of colors used. After elaborate discussions between the three researchers, an agreed‐upon evaluation criterion for the coding of Part B and C was developed, resulting in a high level of consistency among the raters for all coding indicators (ICC > 0.75). The average score of the three raters was taken as the participants' final score.

**TABLE 2:** Coding manual and the cross‐rater agreement.

Coding Category	Subcategory (Indicator)	Instructions or Ratings	Intraclass Correlation Coefficient (ICC)	
			Venting	Distraction
**A**. Color characteristics	a1. Color frequency	Sort all colors used by area size	/	/
	a2. Number of colors	Count the number of colors	/	/
	a3. Dominant color	The dominant color in the picture or the color with the largest area, list only 1–2 kinds	/	/
**B**. Drawing content	b1. Emotion intensity	1 = Extremely weak 5 = Extremely strong	.909***	.855***
	b2. Distinctness of the theme	1 = Not clear at all 5 = Very specific and thematic orientation	.908***	/
	b3. Association between emotion expressed in the drawing and fear	1 = Extremely low 5 = Extremely high	.900***	.872***
	b4. Use of compensation strategy^a^	1 = No 2 = Yes	1.000***	.936***
**C**. Literal description from the drawer	c1. Description of emotion valence while drawing	1 = Extremely negative 5 = Extremely positive	.910***	.905***
	c2. Description of emotion valence expressed in the drawing	1 = Extremely negative 5 = Extremely positive	.852***	.856***
	c3. Richness of description	1 = An incomplete description 5 = Full and rich description	.911***	.873***
	c4. Relation between description and the participant's real life	1 = Not connected at all 5 = Highly connected	.953***	.917***
	c5. Relation between drawing and the video clip	1 = Not connected at all 5 = Highly connected	.869***	.903***
	c6. Association between the description and fear	1 = Extremely low 5 = Extremely high	.907***	.886***

^a^The compensation strategy in b4 refers to the fact that after experiencing negative emotions, some participants deliberately made their drawings safe, serene, or positive to cope with the negative emotions of intense fear.

***p < .001.

## 5 RESULTS

### 5.1 Emotion change during the experiment procedure

#### 5.1.1 Change of valence

**FIGURE 4:**
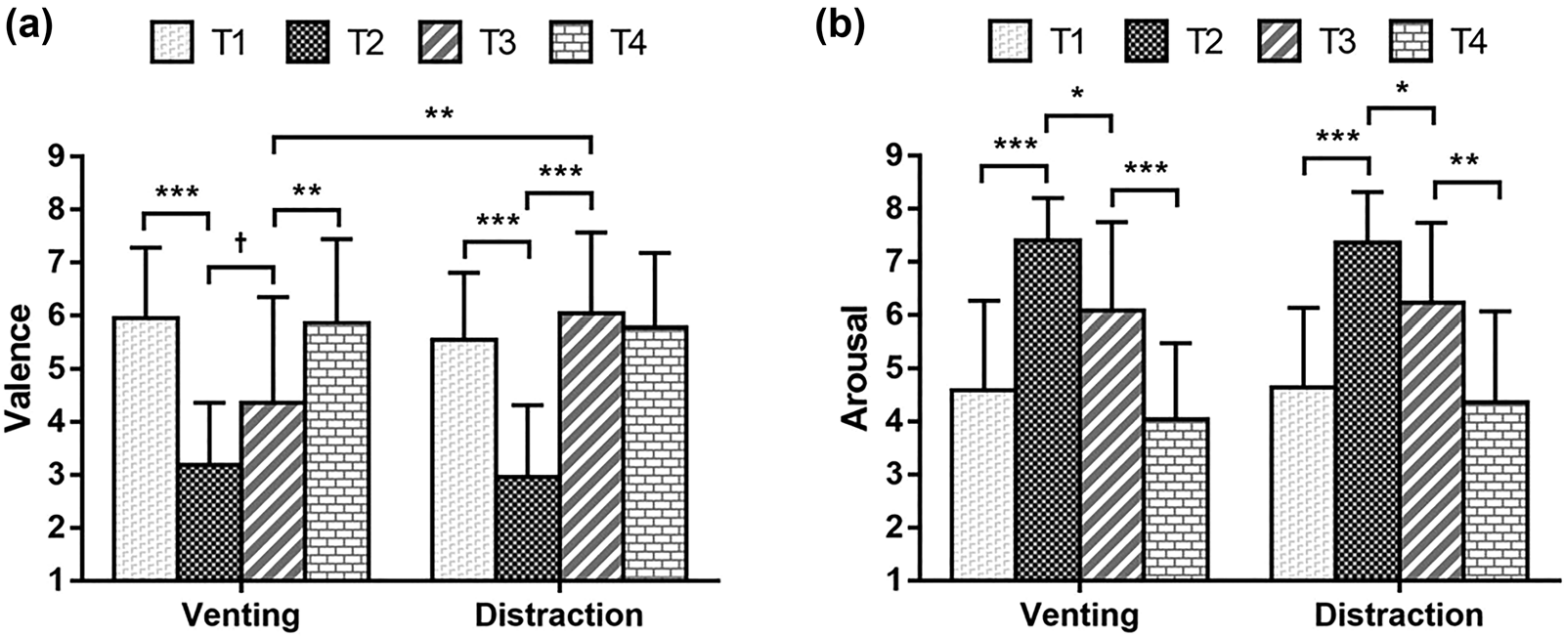
Changing trend of emotion state. *Note*: Part (a) shows the changing trend of the valence of the two groups, and part (b) shows the changing trend of the arousal degree of the two groups. *T*1 was before fear induction, *T*2 was after fear induction, *T*3 was at the end of drawing, and *T*4 was at the end of relaxation. ^†^ *p* < .10; *p < .05; **p < .01; ***p < .001.

The changing trend of the valence of the two groups is shown in Figure 4a A two‐way mixed ANOVA was conducted, in which group (venting, distraction) was the between‐subjects factor and time (*T*1–*T*4) was the within‐subjects factor. The results indicated a significant main effect of time, *F*(3, 126) = 38.229, *p* < .001, *η_p_* ^2^ = .476. Further Bonferroni‐adjusted post hoc comparisons revealed a significant decrease in valence from *T*1 (*M* = 5.75) to *T*2 (*M* = 3.07), *p* < .001, and a significant increase in valence from *T*2 (*M* = 3.07) to *T*3 (*M* = 5.21), *p* < .001. The main effect of group was not significant, *F*(1, 42) = 0.852, *p* = .361, *η_p_* ^2^ = .020. There was a significant interaction between time and group, *F*(3, 126) = 5.406, *p* = .002, *η_p_* ^2^ = .114.

To further clarify the significant interaction, a Bonferroni‐adjusted simple main effect analysis was conducted. Regarding the simple main effects of group, the valence of the distraction group at *T*3 (*M* = 6.05) was significantly higher than that of the venting group (*M* = 4.36), *p* = .003. However, there were no significant differences between the two groups at *T*1, *T*2, and *T*4.

In addition, the simple main effects of time were examined. Valence in the venting group decreased from *T*1 (*M* = 5.96) to *T*2 (*M* = 3.18), *p* < .001, and increased from *T*3 (*M* = 4.36) to *T*4 (*M* = 5.86), *p* = .004. There was no significant difference in valence between *T*2 (*M* = 3.18) and *T*3 (*M* = 4.36), *p* = .062. Valence in the distraction group decreased from *T*1 (*M* = 5.55) to *T*2 (*M* = 2.96), *p* < .001, and increased from *T*2 (*M* = 2.96) to *T*3 (*M* = 6.05), *p* < .001. There was no significant difference in valence between *T*3 (*M* = 6.05) and *T*4 (*M* = 5.77), *p* = 1.000.

#### 5.1.2 Change of arousal

The changing trend of arousal in the two groups is shown in Figure 4b A two‐way mixed ANOVA was conducted, in which group (venting, distraction) was the between‐subjects factor and time (*T*1–*T*4) was the within‐subjects factor. The results indicated a significant main effect of time, *F*(3, 126) = 47.470, *p* < .001, *η_p_* ^2^ = .531. Further Bonferroni‐adjusted post hoc comparisons revealed a significant increase in arousal from *T*1 (*M* = 4.61) to *T*2 (*M* = 7.39), *p* < .001, a significant decrease in arousal from *T*2 (*M* = 7.39) to *T*3 (*M* = 6.16), *p* < .001, and a significant decrease in arousal from *T*3 (*M* = 6.16) to *T*4 (*M* = 4.21), *p* < .001. The main effect of group was not significant, *F*(1, 42) = 0.242, *p* = .625, *η_p_* ^2^ = .006. There was no significant interaction between time and group, *F*(3, 126) = 0.134, *p* = .940, *η_p_* ^2^ = .003.

### 5.2 Drawing coding and its correlation with valence

**FIGURE 5:**
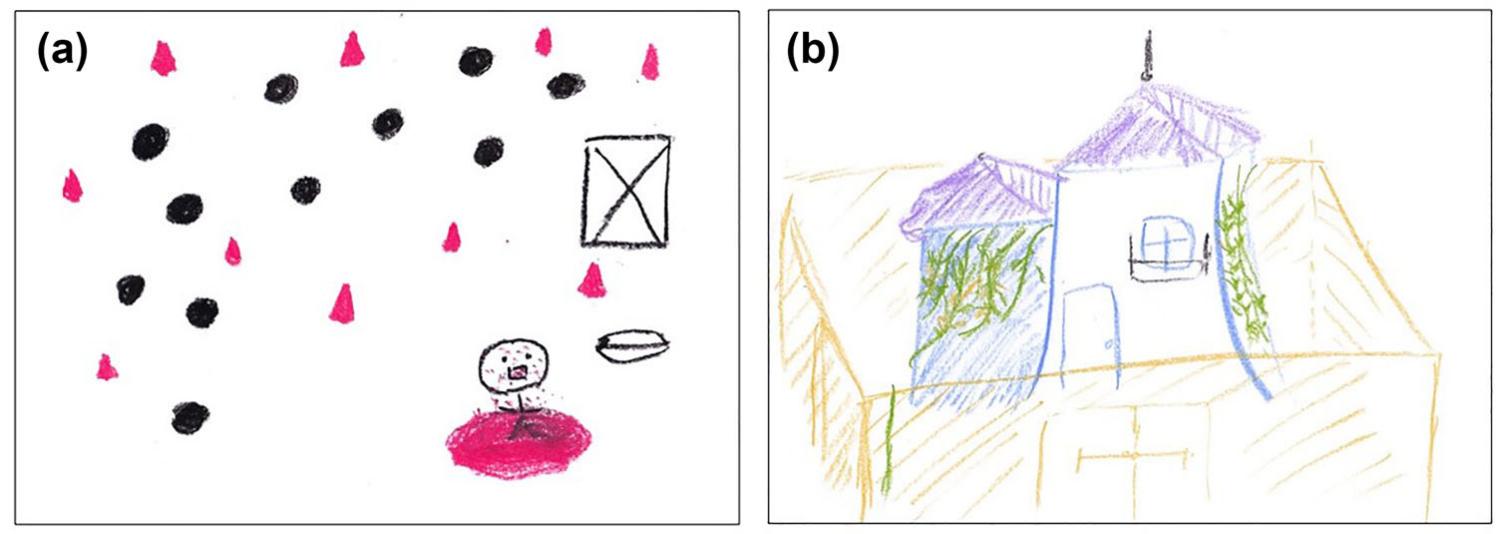
Examples of the drawings. *Note*: Part (a) shows a drawing from the venting group: “The small black circle represents uncertainty; a door that cannot be opened represents no exit and insecurity; a man with his mouth and body full of blood is the poor dead man in the movie; and the blood drops all over the paper represent bloodshed and death.”Part (b) shows a drawing from the distraction group: “An old looking house covered with vines, creepers and other plants. While the main house is still slightly tended, the neighboring house is in a state of disrepair and has been covered with vegetation. The house is surrounded by a wall and the main house has a lightning rod on the roof, which are there because of the desire to protect myself.”

Figure 5 shows specific examples of drawings from the participants in the two groups. The venting drawings were mainly in black (21 participants, 95.5%), red (20 participants, 90.9%), and yellow (11 participants, 50.0%), and the distraction drawings were mainly in blue (19 participants, 86.4%), yellow (18 participants, 81.8%), green (17 participants, 77.3%), and red (13 participants, 59.1%). An independent samples *t*‐test revealed that the number of colors used in distraction drawings (*M* = 4.64, *SD* = 1.00) was significantly higher than that in venting drawings (*M* = 3.45, *SD* = 1.60), *t*(42) = 2.942, *p* = .005, Cohen's *d* = 0.892. This may indicate that participants in the distraction condition tended to be more relaxed and pleasant, whereas the venting group concentrated more on the content and theme.

The descriptive statistics of the coding and the independent‐samples *t*‐test results are provided in Table A1. The venting group scored significantly higher than the distraction group on b1 “Emotion intensity,” b3 “Association between emotion expressed in the drawing and fear,” c5 “Relation between drawing and the video clip,” and c6 “Association between the description and fear,” whereas the distraction group scored significantly higher than the venting group on b4 “Use of compensation strategy,” c1 “Description of emotion valence while drawing” and c2 “Description of emotion valence expressed in the drawing.”

In both conditions, correlation analysis was used between drawing coding and valence at *T*3, variation in valence (denoted as Δ valence) from *T*2 to *T*3, valence at *T*4, and variation in valence from *T*3 to *T*4. The results showed that valence was significantly correlated with indicators b1, c1, and c5 (Table 3). The correlations of all variables are shown in Table A2.

**TABLE 3:** Pearson correlation coefficients between drawing coding and valence.

		*T*3		*T*4	
Indicator	Condition	Valence	ΔValence	Valence	ΔValence
b1. Emotion intensity	Venting	.315	.440*	‐.380	‐.553**
	Distraction	.417	.707***	.098	‐.329
c1. Description of emotion valence while drawing	Venting	.522*	.366	.266	‐.278
	Distraction	.711***	.549**	.345	‐.394
c5. Relation between drawing and the movie clip	Venting	.343	.428*	‐.274	‐.503*
	Distraction	‐.080	‐.143	.361	.417

* *p* < .05.

** *p* < .01.

*** *p* < .001.

As shown in Table 3, the stronger the emotion expressed in the drawings, the greater the emotional improvement after drawing. In the venting condition, the emotion intensity of the drawing was positively correlated with Δ valence at *T*3, *r* = .440, *p* = .040, and negatively correlated with Δ valence at *T*4, *r* = −.553, *p* = .008. In the distraction condition, the emotion intensity of the drawing was positively correlated with Δ valence at *T*3, *r* = .707, *p* < .001.

The more positive the feelings while drawing, the higher the valence after drawing. In the venting condition, positive feelings were positively correlated with valence at *T*3, *r* = .522, *p* = .013. In the distraction condition, positive feelings when drawing were positively correlated with valence at *T*3, *r* = .711, *p* < .001, and Δ valence at *T*3, *r* = .549, *p* = .008.

In the venting condition, the stronger the relationship between the drawings and the film, the greater the improvement in emotions after drawing. The degree of drawing‐film association was positively correlated with Δ valence at *T*3, *r* = .428, *p* = .047, and *T*4, *r* = −.503, *p* = .017.

### 5.3 Activation of prefrontal cortex and its predictive effect on valence

**FIGURE 6:**
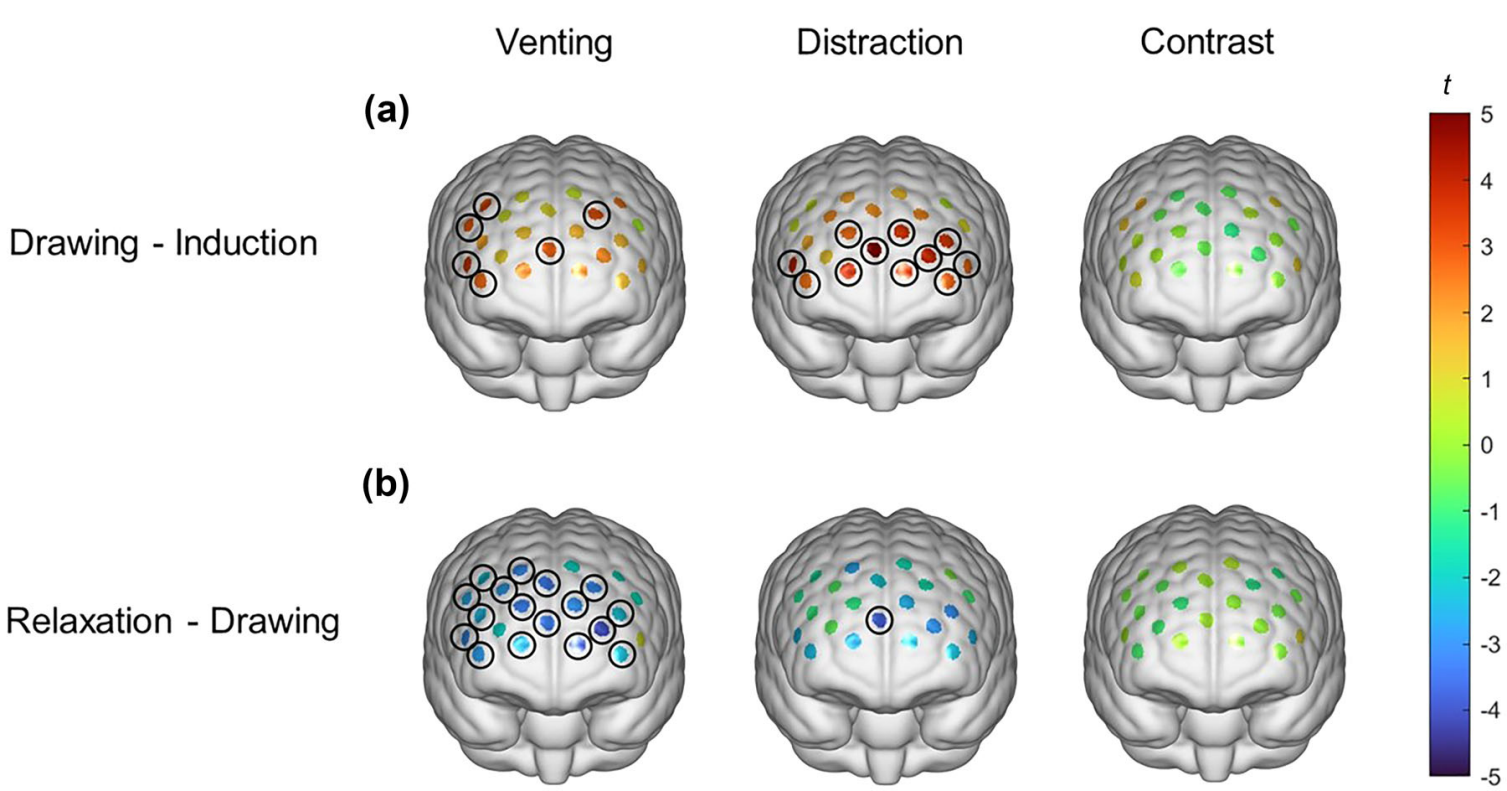
Results of group level statistics. *Note*: Part (a) shows the difference between the drawing and emotion induction periods, and part (b) shows the difference between the relaxation and drawing periods. The left column shows the venting group, the middle column shows the distraction group, and the right column shows the differences between the venting and distraction groups. The significant channels are circled.

Two contrasts were conducted within the groups to show changes in brain activation in the adjacent periods. First, the variation in response values (*β* values) between the drawing and emotion induction periods was calculated. A one‐sample *t*‐test with 0 as the test value revealed positive activation in Channels 4, 7, 9, 15, 18, and 22 in the venting group and positive activation in Channels 1–7 and 9–12 in the distraction group, as shown in Figure 6a. Table A3 shows the statistical results for all channels. In other words, both groups had higher levels of prefrontal activation during the drawing period than during the emotion induction period, and more channels were activated in the distraction group. The independent samples *t*‐test did not reveal any differences between the two groups.

Second, the variation in the response values (*β*) between the relaxation and drawing periods was calculated. A one‐sample *t*‐test with 0 as the test value revealed that the venting group had negative activation in Channels 1–7, 9–17, 21, and 22. The distraction group only had negative activation in Channel 7, as shown in Figure 6b. Table A4 shows the statistical results for all channels. In other words, the overall prefrontal activation level in the venting group was lower in the relaxation period than in the drawing period, whereas the amount of change was smaller in the distraction group. The independent samples *t*‐test did not reveal any differences between the two groups.

**FIGURE 7:**
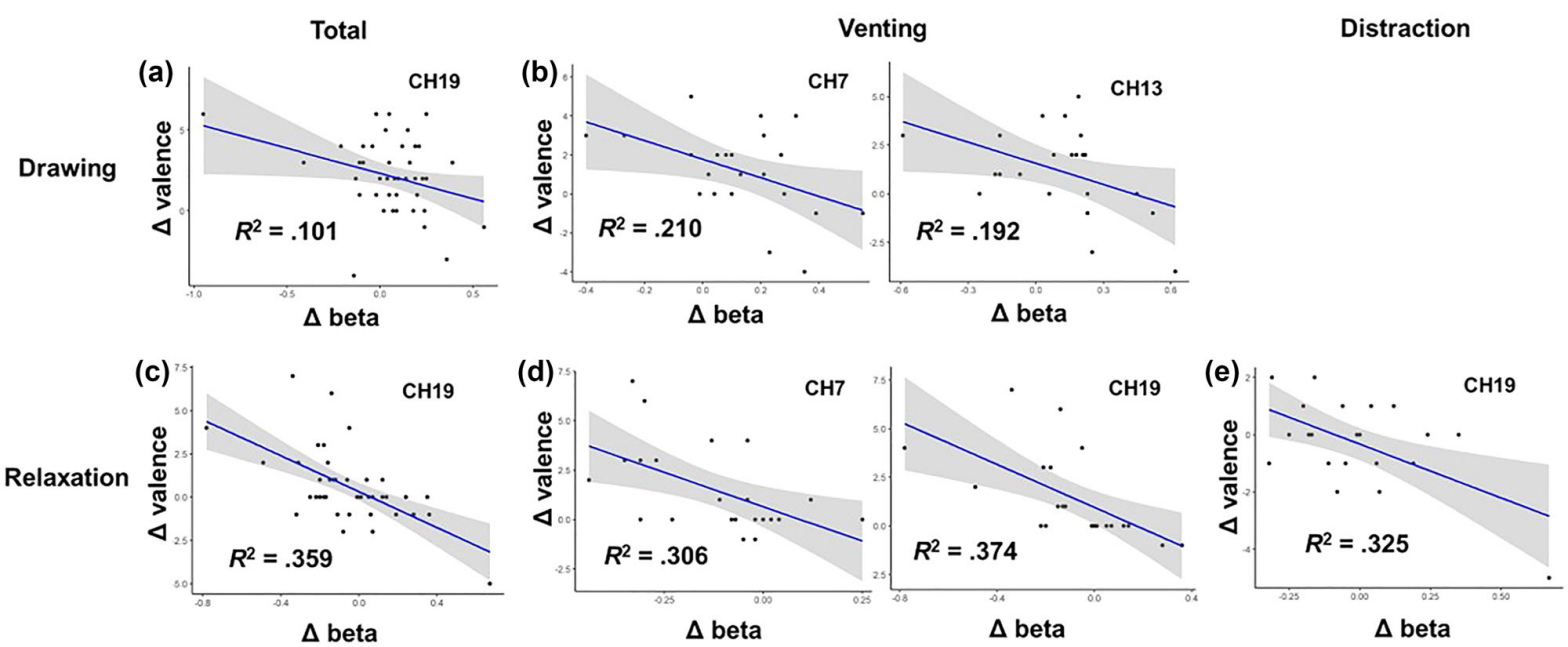
The predictive effects of the response value variation on the valence variation. *Note*: (a) During the drawing period, Δbeta of CH19 in all participants negatively predicted Δ valence; (b) during the drawing period, Δ beta of both CH7 and CH13 in the venting group negatively predicted Δ valence; (c) during the relaxation period, Δ beta of CH19 in all participants negatively predicted Δ valence; (d) during the relaxation period, Δ beta of both CH7 and CH19 in the venting group negatively predicted Δ valence; (e) during the relaxation period, Δ beta of CH19 in the distraction group negatively predicted Δ valence.

For the drawing and relaxation periods, regression analyses were conducted to examine the predictive effect of the variation in the response value of each channel (i.e., the difference between the response value of that period and the previous period, denoted as Δ beta) on the Δ valence of that period. Significant predictive effects were observed for channels 7, 13, and 19, as shown in Table 4 and Figure 7.

**TABLE 4:** The predictive effects of the response value variation on the valence variation.

Period	Group	Channel	*R* ^2^	*B*	β	*t*	*p*
Drawing	Overall	19	.101	−3.123	−.318	−2.171	.036
	Venting	7	.210	−4.809	−.458	−2.302	.032
		13	.192	−3.594	−.438	−2.180	.041
Relaxation	Overall	19	.359	−5.211	−.599	−4.847	***
	Venting	7	.306	−7.087	−.553	−2.972	.008
		19	.374	−5.541	−.612	−3.460	.002
	Distraction	19	.325	−3.743	−.570	−3.106	.006

*** *p* < .001.

## 6 DISCUSSION

The present study examined the sustainability of the differences in the emotion regulation effects of venting and distraction strategies deployed through drawing activity. It explored the relationship among drawing coding, prefrontal fNIRS data, and self‐reported emotion states. The results showed that although the distraction group had a higher valence than the venting group at the end of the drawing activity, there was no difference in valence between the two groups after a brief period of relaxation. Furthermore, prefrontal activity patterns differed between the venting and distraction groups. The number of channels with elevated activity during drawing was greater in the distraction group than in the venting group. In contrast, during relaxation, the number of channels with reduced activity in the venting group was greater than that in the distraction group. Additionally, drawing coding and brain activation data were correlated with self‐reported emotion states.

### 6.1 Sustainability of the difference between venting and distraction strategies

The regulatory effect of drawing on negative emotions is achieved through both venting and distraction mechanisms (De Petrillo & Winner, 2005). Previous research has found that distraction strategies are more conducive to mood repair in the short term (Dalebroux et al., 2008; Diliberto‐Macaluso & Stubblefield, 2015; Drake & Winner, 2012). Most of these studies induced sadness. The present study induced fear and found similar results to previous studies, suggesting that the findings have good generalizability to other negative emotions.

More importantly, the present study added a short relaxation period of less than 4 min at the end of the drawing activity and found that there was no difference in valence between the two groups following this period. This was because the venting group had a significant increase in valence from after drawing to after the relaxation period, whereas valence was maintained at the same level in the distraction group. This finding is a step further than those of previous studies. When people draw to express emotions, they are immersed in the negative emotions induced and naturally have relatively lower valence at the end of drawing. Meanwhile, when people create something neutral or positive to distract attention, valence naturally recovers quicker in a short period. This result is consistent with earlier research on emotion regulation (Kraemer & Hastrup, 1988). The present study found that valence in the venting group improved quickly after a brief period of relaxation, supporting this hypothesis. Future research could also explore how long the difference in effects between the two strategies can be maintained without the relaxation instructions.

The significant correlation between drawing coding and variations in valence also provides evidence to support the positive effect of emotion expression. In the venting group, the stronger the emotion expressed in the drawing (whether positive or negative), and the stronger the association between the drawing and fear, the more their emotion improvement. Moreover, this significant correlation did only occur in the drawing period but also lasted until the next relaxation period. Although expressing emotions caused participants to dwell on negative emotions, those with strong emotional expressions showed greater improvement in valence than those who were relatively calm. In other words, in the venting group, the more intense and thorough the venting, the more it may have contributed to the release of negative emotions and recovery of valence. This is inline with Freud's argument: Emotions are imprisoned in people's hearts, and we have to let them out by catharsis (Freud & Breuer, 1895/1955).

The role of venting has been supported by several studies, either by art‐making or writing. Self‐expression through art‐making can lead to a reduction in the level of the stress hormone cortisol (Kaimal et al., 2016), and expressing emotions through writing can have the same effect (Pennebaker & Smyth, 2016). An early study asked a group of college students to spend half an hour a day for 3–5 days writing down their deepest thoughts and feelings about some very upsetting experiences, whereas the control group wrote about nonemotional topics. At the end‐of‐semester follow‐up, students in the experimental group got sick less, drank less alcohol, and received better grades than the control group (Pennebaker, 1997). Very importantly, the good effects of these activities were not merely due to the expression of emotions. In a study of writing tasks, individuals who frequently used words such as “because,” “why,” and “recognize” benefited more (Pennebaker & Graybeal, 2001). That is to say, they understood the situation better and were more reflective through expression. Conversely, dwelling on emotions is excessive rumination and may cause depression and anxiety (Garnefski et al., 2004; McLaughlin & Nolen‐Hoeksema, 2011).

It can be seen that the effectiveness of venting strategies, whether through art‐making or writing, has been demonstrated in many studies where expression and reflection may work together. The present study demonstrated this again. There was an experimental procedure in the study that triggered self‐reflection in the participants: Both groups were asked to answer six questions after drawing, which in themselves would facilitate participants’ reflection. The coding items of the drawings included the participants’ reflection (Part C “Literal description from the drawer”), and two of the three indicators that significantly correlated with valence came from reflection: c1 “Description of emotion valence while drawing” and c5 “Relation between drawing and the video clip.” It is evident that expression and reflection work together for venting strategies. The role of reflection could be studied specifically in the future.

### 6.2 Insights from fNIRS results

Brain imaging techniques can reveal facts that scales alone cannot reflect. The present study placed the probe patch at the prefrontal cortex. The prefrontal cortex is associated with many functions, but it is generally recognized that it reflects the level of cognitive control (Tisserand et al., 2004). The fNIRS studies of walking and running in the natural state have interpreted prefrontal activation as cognitive load or maintenance of attention (Suzuki et al., 2004, 2008). Some fNIRS studies of art‐making also interpreted prefrontal activation as cognitive control (Kaimal et al., 2022; Yan, Zhang et al., 2021). The present study also adopts such an assumption.

The fNIRS results revealed that the distraction group showed a significant increase in activity on 11 channels during the drawing period, more than the venting group on 6 channels. During the relaxation period, the venting group showed a significant decrease in activity on 18 channels, whereas the distraction group showed a significant decrease on only one channel. If the overall prefrontal activity level is interpreted to reflect the level of cognitive control, the results can easily be explained. Distraction drawing requires more involvement in cognitive control than venting drawing and requires more effort to shift away from intense negative emotions. Meanwhile, although cognitive control was reduced in both groups during the relaxation period, the reduction in cognitive control was more significant in the venting group; in other words, the venting group was relatively more relaxed. It has been argued that using drawing to distract is cognitively demanding, and that such tasks load our working memory and impede mood‐congruent processing, which in turn facilitates the shifting of attention away from negative emotions (Forkosh & Drake, 2017; Van Dillen & Koole, 2007) According to the psychoanalytic school's theory, expressing emotions can release imprisoned energy, and so the venting group was more able to relax. In contrast, repressing emotions requires consuming energy. After the relaxation period, although the valence of the distraction group was similar to that of the venting group, the accumulated energy was still not released, and the cognitive load was still relatively high.

The present study explored the relationship between brain activation data and scale scores and found significant results in three channels: CH7 (covering BA 10, belonging to the mPFC), CH13 (covering mainly BA 10, belonging to the mPFC, and BA 46, belonging to the right dlPFC), and CH19 (covering mainly BA 9 and BA 46, belonging to the left dlPFC), which were mainly present in the venting condition. Interestingly, variations in brain activation levels negatively predicted variations in valence across channels, groups, and periods; lower activation was associated with higher valence, and higher activation was associated with lower valence. This trend may not be coincidental. As the prefrontal activation level reflects the level of cognitive involvement to some extent, the results again coincide with the above findings: The less the cognitive control over emotion and the stronger the free expression of emotion during drawing, the higher the increase in valence; the more the cognitive control over emotion and the less free the expression of emotion, the lower the increase in valence.

It seems that the results of the present study support the superiority of the venting strategy, but it should be noted that distraction and venting both have therapeutic value. Although the long‐term use of distraction strategies can prevent people from taking steps to improve their situation, the arts have been shown to be spontaneously used as a form of distraction or avoidance. According to a recent survey, people were engaged in everyday artistic activities more often during the COVID‐19 pandemic than before, and artistic activities most commonly regulated emotions by providing a means of escape (Drake et al., 2022). The focus of the present study was to point out what has been overlooked in the previous literature. Previous laboratory studies on art‐making have usually concluded that distraction strategies are better than venting strategies, but with fNIRS technology, the advantages of venting can be demonstrated. The results of this study can shed light on practice applications: In time‐limited situations, therapists can use distraction strategies to enable clients to regulate their emotions quickly; however, venting strategies can also achieve good results if there is enough time and space. In this way, art therapists can choose appropriate strategies for the situation of the scene, making clinical practice evidence‐based.

The groupings in this study can be interpreted from two perspectives. One group in this study drew the emotions felt while watching the video, and the other drew a house. From the perspective of emotion regulation, this can be interpreted as venting and distraction. In terms of the content of the drawings, the former is an expression of emotion, and the latter is a neutral object. Therefore, a comparison between the two groups can help to understand whether brain activity varies depending on the content of the drawing. Although this study found some differences in the brain activation results between the two groups, independent sample *t*‐tests did not show significant between‐group differences. This implies that brain activation is similar in the same form of drawing, regardless of content. Therefore, this provides another explanation for the study conducted by Yan, Ji et al. (2021): The difference in brain activation between people with schizophrenia and healthy controls when creating emotional drawings may not be due to differences in emotion expression, but rather due to the drawing activity itself (Nakano et al., 2018).

### 6.3 Limitations and prospects

This study had some limitations. Raising these issues would be beneficial for future research. The first was the interpretation of the fNIRS activation results. In fact, scholars have not yet agreed upon a definite interpretation of brain activation. For example, some studies have suggested that negative activation of the mPFC reflects negative emotions (Huang et al., 2017). However, the present study found a decrease in mPFC activity during the relaxation period with an increase in valence, which is not in line with the above hypothesis. In this study, we considered the prefrontal cortex as a whole and regarded the activation level of the prefrontal cortex to generally reflect the level of cognitive control. This interpretation can make the results of self‐reported emotion, drawing coding, and fNIRS data corroborate with each other. However, we do not exclude other possibilities. Future studies could provide more evidence by using other measures, such as asking participants directly how “relaxed” they feel, or by employing other indicators, such as skin electricity.

The second issue was the manipulation of the experiment. The manipulation in this experiment was achieved through different instructions, defining expressing emotions as venting and drawing something else as distraction. However, the experiences of some participants may not have been as expected from the experimental manipulation. In this study, the subjective report questionnaire was designed to ask whether the content of the drawing was related to the emotions experienced while watching the movie (c5). It was assumed that the venting group would report that it was related, and the distraction group would report that it was not. However, participants in the distraction group also reported some degree of relation (*M* = 3.08, see Table A1), suggesting that even when given the distraction task, people were not completely unaffected by the previously induced emotion. Moreover, the coding of compensation strategies (b4) indicated that the distraction group used more compensation strategies than the venting group. It was because participants in the distraction group experienced strong fearful emotions while viewing the film that they deliberately depicted peace, safety, and comfort in the drawing to cope with the fearful emotions. This suggests that, regardless of venting or distraction, the underlying working mechanisms may have commonalities, such as expressing emotions directly or indirectly. Nevertheless, the present study could not identify such commonalities nor were they mentioned or explored in previous studies.

Third, although this study examined the sustainability of the difference between venting and distraction strategies, it was still a short period lasting less than four minutes. By analyzing data from different sources, this study revealed the advantages of the venting strategy to some extent. However, more convincing results should be directly reflected in explicit indicators. Future studies could extend the duration of observation, such as setting other unrelated tasks after the relaxation period and measuring various indicators after the additional task. This approach may bring more insight into the difference between venting and distraction strategies.

## 7 CONCLUSION

After the induction of fear, distraction drawing was more conducive to rapid emotion recovery than venting drawing, but the valence of the venting group improved after a relaxation period, resulting in no difference between groups. Further fNIRS data revealed that, compared to the distraction group, the venting group had fewer channels with elevated prefrontal activity during drawing, suggesting less cognitive control, and more channels with reduced prefrontal activity during relaxation, suggesting a higher level of relaxation. The drawing coding and fNIRS data were associated with variations in valence. Therefore, it can be interpreted that the less the cognitive control over emotion and the more free the expression of emotion during drawing, the higher the increase in valence; the more the cognitive control over emotion and the less free the expression of emotion, the lower the increase in valence.

## AUTHOR CONTRIBUTIONS

Wenhua Yan contributed to the overall scheduling, research design, material preparation, and the development of the drawing coding system. Xinlei Zhang and Cheng Xu contributed to the study design, material preparation, data collection, and analysis. Aiping Yang, Zihan Shen, and Xinwei Guo contributed to data collection and drawing coding. The first draft of the manuscript was written by Xinlei Zhang and all authors commented on previous versions of the manuscript. All authors read and approved the final manuscript.

## CONFLICT OF INTEREST STATEMENT

The authors declare that they have no conflicts of interest.

## FUNDING INFORMATION

The authors declare that no financial support was received for the research, authorship, and/or publication of this article.

### PEER REVIEW

The peer review history for this article is available at https://publons.com/publon/10.1002/brb3.3248.

## Supporting information


**Table A1** Independent‐samples *t*‐tests of drawing coding between the two conditions.
**Table A2** Pearson correlation coefficients between drawing coding and valence.
**Table A3** One‐sample *t*‐tests on Δ*β* values of the drawing phase (FDR corrected).
**Table A4** One‐sample *t*‐tests on Δ*β* values of the relaxation phase (FDR corrected).

## Data Availability

The raw data supporting the conclusions of this article can be obtained by emailing: whyan@psy.ecnu.edu.cn.
