# Supplementary material for: Functional near‐infrared spectroscopy approach to the emotional regulation effect of drawing: Venting versus distraction
Source: Brain Behav. 2023 Sep 12;13(11):e3248. doi: 10.1002/brb3.3248 (PMC10636421; doi:10.1002/brb3.3248)
Supplement: Supplementary file 1 — Table A1 Independent‐samples t‐tests of drawing coding between the two conditions. Table A2 Pearson correlation coefficients between drawing coding and valence. Table A3 One‐sample t‐tests on Δβ values of the drawing phase (FDR corrected). Table A4 One‐sample t‐tests on Δβ values of the relaxation phase (FDR corrected). [file BRB3-13-e3248-s001.docx]

**Supporting information**

**Table A1**

*Independent-samples T-test of Drawing Coding Between the Two Conditions*

| Coding category | Venting (*M*±*SD*) | Distraction (*M*±*SD*) | *t* | Cohen’s *d* |
| --- | --- | --- | --- | --- |
| b1. Emotion intensity | 4.36±0.97 | 3.00±1.15 | **4.261***** | 1.278 |
| b2. Distinctness of the theme | 3.29±1.52 | / | / | / |
| b3. Association between emotion expressed in the drawing and fear | 3.89±1.30 | 1.45±0.57 | **8.071***** | 2.431 |
| b4. Use of compensation strategy | 1.09±0.29 | 1.65±0.48 | **-4.694***** | -1.412 |
| c1. Description of emotion valence while drawing | 2.41±1.19 | 3.70±1.15 | **-3.636***** | -1.102 |
| c2. Description of emotion valence expressed in the drawing | 2.33±0.74 | 3.30±0.53 | **-4.977***** | -1.507 |
| c3. Richness of description | 3.17±1.26 | 2.92±0.94 | 0.723 | 0.225 |
| c4. Relation between description and the participant’s real life | 2.83±1.68 | 3.24±1.60 | -0.827 | -0.250 |
| c5. Relation between drawing and the video clip | 4.70±0.80 | 3.08±1.71 | **4.022***** | 1.214 |
| c6. Association between the description and fear | 3.59±1.46 | 2.23±1.11 | **3.490**** | 1.049 |

Note. * *p* < .05; ** *p* < .01; *** *p* < .001

**Table A2**

*Correlation Coefficients Between Drawing Coding and Valence*

| Coding category | Condition | T3 | |  | T4 | |
| --- | --- | --- | --- | --- | --- | --- |
|  |  | Valence | ΔValence |  | Valence | ΔValence |
| b1. Emotion intensity | Venting | .315 | **.440*** |  | -.380 | **-.553**** |
|  | Distraction | .417 | **.707***** |  | .098 | -.329 |
| b2. Distinctness of the theme | Venting | .132 | .214 |  | .050 | -.082 |
|  | Distraction | / | / |  | / | / |
| b3. Association between emotion expressed in the drawing and fear | Venting | -.095 | .101 |  | -.224 | -.074 |
|  | Distraction | .249 | .357 |  | -.122 | -.365 |
| b4. Use of compensation strategy | Venting | -.141 | -.027 |  | -.074 | .073 |
|  | Distraction | -.064 | -.068 |  | .348 | .389 |
| c1. Description of emotion valence while drawing | Venting | **.522*** | .366 |  | .266 | -.278 |
|  | Distraction | **.711***** | **.549**** |  | .345 | -.394 |
| c2. Description of emotion valence expressed in the drawing | Venting | -.011 | .087 |  | -.203 | -.135 |
|  | Distraction | -.232 | -.028 |  | .054 | .283 |
| c3. Richness of description | Venting | .253 | .085 |  | .274 | -.031 |
|  | Distraction | -.208 | .004 |  | -.062 | .152 |
| c4. Relation between description and the participant’s real life | Venting | .029 | -.145 |  | .087 | .036 |
|  | Distraction | .177 | .216 |  | .369 | .165 |
| c5. Relation between drawing and the video clip | Venting | .343 | **.428*** |  | -.274 | **-.503*** |
|  | Distraction | -.080 | -.143 |  | .361 | .417 |
| c6. Association between the description and fear | Venting | .004 | .200 |  | -.313 | -.227 |
|  | Distraction | -.383 | -.101 |  | -.077 | .313 |

Note. * *p* < .05; ** *p* < .01; *** *p* < .001

**Table A3**

*One-sample T-tests on Δ β Values of the Drawing Phase (FDR corrected)*

| Channel | Venting Group | | | Distraction Group | | |
| --- | --- | --- | --- | --- | --- | --- |
|  | Δ *β* (*M*±*SD*) | *t* | *p* | Δ *β* (*M*±*SD*) | *t* | *p* |
| 1 | 0.08±0.27 | 1.483 | .153 | 0.09±0.16 | 2.796 | **.011*** |
| 2 | 0.11±0.23 | 2.370 | .027 | 0.13±0.17 | 3.658 | **.001*** |
| 3 | 0.11±0.23 | 2.318 | .031 | 0.14±0.18 | 3.795 | **.001*** |
| 4 | 0.15±0.25 | 2.727 | **.013*** | 0.09±0.16 | 2.617 | **.016*** |
| 5 | 0.47±1.42 | 1.562 | .133 | 0.09±0.16 | 2.620 | **.016*** |
| 6 | 0.09±0.18 | 2.258 | .035 | 0.14±0.16 | 3.983 | **.001*** |
| 7 | 0.13±0.21 | 2.799 | **.011*** | 0.15±0.13 | 5.476 | **.000*** |
| 8 | 0.06±0.24 | 1.175 | .253 | 0.05±0.16 | 1.310 | .204 |
| 9 | 0.16±0.23 | 3.262 | **.004*** | 0.13±0.17 | 3.696 | **.001*** |
| 10 | 0.14±0.43 | 1.534 | .140 | 0.15±0.20 | 3.492 | **.002*** |
| 11 | 0.06±0.19 | 1.569 | .132 | 0.13±0.17 | 3.585 | **.002*** |
| 12 | 0.07±0.24 | 1.433 | .167 | 0.08±0.14 | 2.653 | **.015*** |
| 13 | 0.11±0.27 | 1.858 | .077 | 0.08±0.22 | 1.735 | .097 |
| 14 | 0.02±0.41 | 0.215 | .832 | 0.08±0.38 | 0.947 | .354 |
| 15 | 0.08±0.12 | 3.002 | **.007*** | 0.07±0.14 | 2.233 | .037 |
| 16 | 0.05±0.23 | 1.009 | .325 | 0.11±0.24 | 2.081 | .050 |
| 17 | 0.03±0.20 | 0.757 | .458 | 0.06±0.13 | 2.170 | .042 |
| 18 | 0.19±0.31 | 2.831 | **.010*** | 0.05±0.33 | 0.740 | .468 |
| 19 | 0.10±0.19 | 2.454 | .023 | 0.02±0.26 | 0.378 | .709 |
| 20 | 0.03±0.25 | 0.616 | .544 | 0.09±0.28 | 1.450 | .162 |
| 21 | 0.04±0.24 | 0.822 | .420 | 0.11±0.28 | 1.833 | .081 |
| 22 | 0.14±0.22 | 3.032 | **.006*** | 0.03±0.23 | 0.594 | .559 |

Note. * Significant after FDR correction.

**Table A4**

*One-sample T-tests on Δ β Values of the Relaxation Phase (FDR corrected)*

| Channel | Venting Group | | | Distraction Group | | |
| --- | --- | --- | --- | --- | --- | --- |
|  | Δ *β* (*M*±*SD*) | *t* | *p* | Δ *β* (*M*±*SD*) | *t* | *p* |
| 1 | -0.11±0.22 | -2.266 | **.034*** | -0.09±0.19 | -2.302 | .032 |
| 2 | -0.15±0.18 | -3.866 | **.001*** | -0.13±0.26 | -2.360 | .028 |
| 3 | -0.11±0.20 | -2.522 | **.020*** | -0.11±0.21 | -2.547 | .019 |
| 4 | -0.12±0.20 | -2.776 | **.011*** | -0.07±0.14 | -2.406 | .025 |
| 5 | 0.47±2.68 | 0.824 | .419 | -0.10±0.19 | -2.470 | .022 |
| 6 | -0.12±0.13 | -4.085 | **.001*** | -0.11±0.18 | -2.926 | .008 |
| 7 | -0.12±0.17 | -3.256 | **.004*** | -0.13±0.16 | -3.820 | **.001*** |
| 8 | -0.08±0.19 | -1.993 | .059 | -0.03±0.20 | -0.627 | .538 |
| 9 | -0.11±0.17 | -3.017 | **.007*** | -0.12±0.23 | -2.453 | .023 |
| 10 | -0.17±0.34 | -2.283 | **.033*** | -0.13±0.21 | -2.977 | .007 |
| 11 | -0.11±0.17 | -2.946 | **.008*** | -0.10±0.19 | -2.558 | .018 |
| 12 | -0.11±0.16 | -3.242 | **.004*** | -0.05±0.21 | -1.160 | .259 |
| 13 | -0.11±0.21 | -2.423 | **.024*** | -0.07±0.29 | -1.087 | .289 |
| 14 | -0.13±0.34 | -1.784 | .089 | -0.08±0.39 | -0.974 | .341 |
| 15 | -0.10±0.16 | -3.061 | **.006*** | -0.05±0.14 | -1.608 | .123 |
| 16 | -0.10±0.15 | -3.330 | **.003*** | -0.11±0.24 | -2.171 | .042 |
| 17 | -0.07±0.13 | -2.779 | **.011*** | -0.04±0.23 | -0.770 | .450 |
| 18 | -0.13±0.22 | -2.652 | **.015*** | -0.10±0.36 | -1.268 | .219 |
| 19 | -0.10±0.25 | -1.895 | .072 | -0.02±0.23 | -0.307 | .762 |
| 20 | -0.07±0.16 | -1.934 | .067 | -0.09±0.26 | -1.697 | .104 |
| 21 | -0.12±0.18 | -3.066 | **.006*** | -0.14±0.24 | -2.697 | .013 |
| 22 | -0.09±0.19 | -2.263 | **.034*** | -0.08±0.24 | -1.479 | .154 |

Note. * Significant after FDR correction.
